# Antioxidant Activity of *Passiflora edulis* Sims Leaves

**DOI:** 10.4103/0250-474X.56038

**Published:** 2009

**Authors:** M. Sunitha, K. Devaki

**Affiliations:** Department of Biochemistry, Karpagam Arts and Science College, Coimbatore-641 021, India

**Keywords:** Antioxidant, DPPH assay, reducing power activity, Passiflora edulis

## Abstract

Ethanol extract of *Passiflora edulis* Sims was analyzed for its antioxidant (1,1-diphenyl-2-picryl hydrazyl radical reducing power methods) and phytochemical analysis. The extract was found effective against the antioxidant test models exhibiting an IC_50_ value of 875±87.83 μg/ml and showed strong potential antioxidant activity in both assays.

*Passiflora edulis* Sims (Passifloraceae) is a woody climber, native of Brazil, now cultivated in all parts of the world, chiefly for its edible fruits and for its ornamental flowers. The plant is commonly called as yellow passion fruit, *maracuja*, yellow granadilia, and *pomme liane jaune*. In traditional system of medicine, *P. edulis* is used as sedative, antiasthmatic and emetic[1].*P. edulis* leaves are used in the treatment of insomnia and traditionally known to produce a restful sleep without any narcotic hangover. The leaves are reported to contain a bitter principle maracugine, resins, acids and tannin exceptionally rich in ascorbic acid. It is also used to treat epilepsy, ulcers and haemorrhoids[2]. The present investigation was undertaken to evaluate the antioxidant activity of leaf extracts of *P. edulis*.

Leaves of the plant were collected from Coimbatore and identified at the Botanical Survey of India, Coimbatore. The leaves were shade dried and powdered. They were exhaustively extracted in Soxhlet apparatus with ethanol. Preliminary phytochemical screening was carried out[3] and presented in Table 1. Antioxidant activity of the plant extracts was studied by 1,1-diphenyl-2-picryl hydrazyl radical (DPPH) quenching assay and reducing power test models.*In vitro* DPPH radical scavenging activity was carried out by adopting the method of Blois[4]. Different concentrations of the extracts (1000, 500, 250, 125, 62.5, 31.25 μg/ml) were prepared and subjected to antioxidant tests. To 500 μl of each of the extracts, 5 ml of 0.1 mM methanol solution of DPPH was added, vortexed, followed by incubation at 27° for 20 min. The control was prepared without any extract and absorbance of the sample was measured at 517 nm using UV/Vis spectrophotometer. Radical scavenging activity was expressed as percentage inhibition of DPPH radicals.

**TABLE 1:** PHYTOCHEMICAL ANALYSIS OF *P. EDULIS*

Chemical components	Leaf extracts
Alkaloids	−
Saponin	+
Tannin and Phenolic compounds	+
Flavonoids	+
Steroids	+
Oils and Fats	+
Terpenoids	+

“+” indicates presence of compounds;

“−” denotes absence of compounds

IC_50_ value was also calculated, ascorbic acid was used as the reference standard. The reducing power of the ethanol extract was carried out by adapting the method of Oyaizu[5]. Different concentrations of the extracts (1000, 500, 250, 125, 62.5, 31.25 μg/ml) were prepared. To all the test tubes 2.5 ml of sodium phosphate buffer followed by 2.5 ml of 1% potassium ferrocyanide solution was added. The contents were vortexed well and then incubated at 50° for 20 min. After incubation, 2.5 ml of 10 % trichloroacetic acid was added to all the tubes and centrifugation was carried out at 3000 rpm for 10 min. To 5 ml of the supernatant, 5 ml of distilled water was added. To this about 1 ml of 1% ferric chloride was added to each test tube and incubated at 35° for 10 min. The absorbance was read at 700 nm. The reducing power of the extract was linearly proportional to the concentration of the sample. Ascorbic acid was taken as reference standard. The result of phytochemical analysis was recorded in Table 1. The leaf extract of *P. edulis* exhibited potential antioxidant activity in the both the assay models (Tables 2 and 3)

**TABLE 2:** FREE RADICAL SCAVENGING ACTIVITY OF *P. EDULIS* BY DPPH RADICAL INHIBITION

Concentration (μg/ml)	Inhibition (%) Mean±Std^a^	IC _50_ (μg/ml) Mean±Std^a^
1000	58.17±2.45	875±87.83
500	35.01±1.42	
250	25.53±6.25	
125	15.24±6.72	
62.5	10.81±6.43	
31.25	7.06±3.87	
Ascorbic acid	11.24±0.023	

a denotes Mean±STD at 95% Confidence Interval

**TABLE 3:** REDUCING POWER ACTIVITY OF *P. EDULIS*

Concentration (μg/ml)	Absorbance at 700nm Mean±Std^a^
1000	0.750
500	0.612
250	0.562
125	0.440
62.5	0.331
31.25	0.217
Control (BHA)	0.076
Ascorbic acid (1000μg/ml)	1.701

a denotes Mean±STD at 95% Confidence Interval
